# Proteomics analysis of chicken peripheral blood lymphocyte in Taishan *Pinus massoniana* pollen polysaccharide regulation

**DOI:** 10.1371/journal.pone.0208314

**Published:** 2018-11-29

**Authors:** Shifa Yang, Zengcheng Zhao, Anyuan Zhang, Fengjuan Jia, Minxun Song, Zhongli Huang, Jian Fu, Guiming Li, Shuqian Lin

**Affiliations:** 1 Institute of Poultry Science, Shandong Academy of Agricultural Science, Jinan, Shandong, China; 2 Shandong Provincial Key Laboratory of Poultry Diseases Diagnosis and Immunology, Jinan, Shandong, China; 3 Institute of Veterinary Drug Qualily Inspection of Shandong Province, Jinan, Shandong, China; 4 Institute of Agro-Food Science and Technology, Shandong Academy of Agricultural Sciences, Jinan, Shandong, China; Universidad Miguel Hernández de Elche, SPAIN

## Abstract

The natural polysaccharides extracted from the pollen of *Pinus massoniana* (TPPPS) have been shown to be a promising immune adjuvant against several viral chicken diseases. However, the exact mechanism through which TPPPS enhances the host immune response in chicken remains poorly understood. In the current study, chicken peripheral blood lymphocytes were treated with varying concentrations of TPPPS and pro-inflammatory cytokines such as IFN-γ, iIL-2 and IL-6 were measured to determine the optimal dose of the polysaccharide. A comparative analysis was subsequently performed between the proteome of lymphocytes subjected to the best treatment conditions and that of untreated cells. Protein identification and quantitation revealed a panel of three up-regulated and seven down-regulated candidates in TPPPS-treated chicken peripheral blood lymphocytes. Further annotation and functional analysis suggested that a number of those protein candidates were involved in the regulation of host innate immune response, inflammation and other immune-related pathways. We believe that our results could serve as a stepping stone for further research on the immune-enhancing properties of TPPPS and other polysaccharide-based immune adjuvants.

## Introduction

Polysaccharides are structurally complex macromolecules with a wide range of biological functions, including the mediation of signal transduction, antibody-antigen recognition, bacterial invasion, etc. [1]. Recently, plant- and fungus-derived polysaccharides have attracted significant attention due to their low toxicity, excellent biocompatibility and potential therapeutic values [2]. For example, these carbohydrate macromolecules have been used to construct tissue engineering scaffolds, which demonstrated controllable degradability and excellent mechanical strength. Polysaccharides from several mushroom species have shown clinical potential for stimulating anti-tumor immune response [3]. Plant and fungal polysaccharides have also been employed as natural adjuvants in vaccines as a result of their immunopotentiating properties [4,5]. *Pinus massoniana* is the principal pine species in the Taishan area of China. The natural polysaccharides extracted from its pollens (TPPPS) have previously been shown to promote immune response to several viral diseases in rabbits, chickens and mice [6–8]. Despite these findings, the exact mechanism underlying the immunostimulatory properties of TPPPS remains poorly understood.

During the last several decades, proteomics has emerged as a powerful technology for comprehensively characterizing all proteins in complex biological systems. One obvious advantage of proteomics over genomics lies in its ability to capture critical information that is unavailable on the DNA level, such as protein-protein interaction and post-translational modification. Therefore, proteomic data are considered to more accurately reflect the activities of various pathways and the overall status of the cell. The proteomic approach has been employed to elucidate the mechanisms and identify the biomarkers of diseases. For example, Van Altena et al. has recently shown by proteomic profiling that 13 proteins exhibited enhanced expression in the milk of dairy cows with increased susceptibiltiy to bovine diseases [9]. In another study, Genini and coworkers described a panel of 14 differentially expressed proteins that could be used to distinguish piglets with porcine reproductive and respiratory syndrome from healthy controls with a sensitivity and specificity of 77% and 73%, respectively [10]. These studies highlighted the utility of proteomics in helping researchers interpret various complex pathophysiological processes on a molecular level.

In the current study, we comprehensively analyzed the proteome of peripheral blood lymphocytes in chickens administered with TPPPS to investigate the immunostimulatory effects of the polysaccharides. Bioinformatic analysis of the identified proteins revealed a panel of 10 differentially expressed candidates. Subsequent functional analysis suggested that a number of those proteins were involved in the regulation of host innate immune response, inflammation and other immune-related pathways. We believe that our study could serve as a stepping stone for further research on the immune-enhancing properties of TPPPS and other polysaccharide-based immune adjuvants.

## Materials and methods

### Ethics statement

All animal procedures performed in this study were reviewed, approved, and supervised by the Animal Ethics committee of Shandong Academy of Agricultural Science (Permit No.: 2017412).

### Isolation and cultivation of chicken peripheral blood lymphocytes

Adult specific pathogen-free chickens were used and housed under conventional conditions. The ambient conditions for the studies were set at 20 to 25°C and 30% to 40% relative humidity, and the air entering the isolator was filtered. To alleviate pain, each chicken received an intramuscular injection of 0.2 mL/kg xylazine hydrochloride ten minutes prior to the blood withdrawal. One milliliter of blood was drawn from the wing vein into a sterile collector tube containing 0.2 mL of 3.8% sodium citrate as an anticoagulant. The resulting mixture was carefully layered on top of 2 mL of Ficoll-hypaque solution, followed by centrifugation at 1500 rpm for 15 min. Four layers were visible from top to bottom after centrifugation, which included a plasma layer, a white lymphocyte layer, a transparent Ficoll-hypaque layer and an erythrocyte layer. Therefore, the top plasma layer was carefully removed and the second layer of white lymphocytes was collected via a sterile pipette tip into a centrifuge tube containing 5 mL phosphate buffered saline (PBS). After thorough mixing, the tube was centrifuged at 1500 rpm for 20 min. Cell pellet was washed twice with PBS. Viability of the harvested white lymphocytes was evaluated using Trypan Blue following a previously described method [11]. The cells were then diluted in RPMI-1640 supplemented with 10% fetal bovine serum (FBS) to a final concentration of 1×10^6^ mL^-1^. The cell samples were enriched in lymphocytes but also contained other white cells such as monocytes and platelets. Then, 2 mL of the cell suspension was added to each well of 6-well plates and cultured at 37 ^o^C under 5% CO_2_.

### Preparation of TPPPS

TPPPS was extracted from the pollen of Taishan Pinus massoniana based on a previously established protocol [6,12]. Briefly, the wall-broken pollens were first extracted with ethyl ether in a Soxhelt extractor to remove the lipid content. The lipid-free pollens were then mixed with 25 vol of deionized water containing 0.3% of trypsin and the pH of the resultant liquid mixture was adjusted to 8.0 by NaOH. After incubation at 50 ^o^C for 2 h, the supernatant was collected by centrifugation and the undissolved materials were re-extracted with the abovementioned cellulase solution. The pollens were extracted for a total of four times to maximize the yield of TPPPS. The supernatants from all four extractions were pooled, filtered, centrifuged at 10000 rpm and then concentrated under reduced pressure by rotary evaporation. The protein contaminants were removed from the TPPPS concentrate by treatment with sevage reagent twice. Purified TPPPS was obtained from the protein-free concentrate by precipitating with 4 vol of anhydrous ethanol. The polysaccharide precipitates were then collected and freeze-dried. The purity of the obtained TPPPS was analyzed by phonel-sulfate methodand was shown to be 94.9%.

### TPPPS treatment

After two hours of incubation, the culture supernatant was carefully removed and 2 mL of fresh RPMI-1640 medium containing 10% FBS and TPPPS was added to each well to one of the following final concentrations, including 0, 12.5, 50, 100, 200, 400, 800, 1600 μg/mL. The culture was then incubated under the same conditions for an additional 16–48 h until the cells reached confluency. The supernatant was then carefully transferred to a fresh centrifuge tube. The level of interferon (IFN)-γ, interleukin (IL)-2 and IL-6 was quantified using Chicken IFN-γ ELISA Kit (Mlbio, Shanghai, China) based on the manufacturer’s instructions. The optimal TPPPS concentration was determined based on the expression levels of the above cytokines and was subsequently used for studying the proteomic changes in chicken peripheral blood lymphocytes.

### Vitality assay

The peripheral lymphocyte treated with TPPPS were incubated for 24 h at 37°C, and 10 μL of CCK-8 solution (BOSTER, China) was added to each well. The absorbance at 450 nm was measured on a microplate reader. The Peripheral lymphocyte vitality was calculated based on the OD450.

### Sample preparation

Roughly 5 × 10^6^ chicken peripheral blood lymphocytes were washed three times with PBS and then suspended in 100 μl of lysis buffer (2 M thiourea, 7 M Urea, 4% 3-[(3-cholamidopropyl) dimethylammonio] -1- propanesulfonate, 40 mM Tris-HCl, pH 8.5) supplemented with 1 mM phenylmethylsulfonyl fluoride and 2 mM ethylenediaminetetraacetic acid. After 5 min of incubation at 4 ^o^C, dithiothreitol (DTT) was added to a final concentration of 10 mM. The cell suspension was sonicated for 15 min and then centrifuged at 25000 g and 4 ^o^C for 20 min. Proteins were precipitated by mixing the supernatant with 5 vol of pre-chilled acetone at -20 ^o^C, followed by incubation at the same temperature for 2 h. The resultant mixture was then centrifuged at 16000 g for 20 min and the supernatant was discarded. The pellet was re-suspended in the same aforementioned lysis buffer, followed by the addition of DTT, sonication and centrifugation using the identical procedures described above. After the centrifugation, the supernatant was warmed to 56 ^o^C and treated with a final concentration of 10 mM DTT for 1 h to reduce all disulfide bonds. Alkylation of cysteine was performed by adding iodoacetamide to a final concentration of 55 mM and incubating the resultant solution in darkness for 45 min at 4 ^o^C. The proteins were pelleted again by adding pre-chilled acetone, incubating at -20 ^o^C for 2 h, and centrifuging at 25000 g for 20 min. The pellet was dissolved by sonication in 200 μL of 0.5 M triethylammonium bicarbonate (TEAB) buffer for 15 min at 4 ^o^C and then centrifuged at 25000 g for 20 min. The supernatant was collected and analyzed by sodium dodecyl sulfate polyacrylamide gel electrophoresis as well as Bradford assay.

### Trypsin digestion and iTRAQ labeling

Based on the quantitation results, 100 μg of proteins were mixed with 5 μg of trypsin (MERCK, Germany) in a total volume of 100 μl, which was then incubated at 37 ^o^C for 16 h. Another 5 μg of trypsin was added and the solution was incubated at 37 ^o^C for an additional 8 h. iTRAQ labeling was performed based on previous described protocols with minor modifications [13]. Briefly, the trypsin-digested peptides were using vacuum centrifugation. The peptides were re-dissolved in 0.5 M TEAB buffer and subjected to iTRAQ labeling. In brief, the iTRAQ reagents (SCIEX, USA) were reconstituted in isopropanol to a final concentration of 20 mM. The peptides were then incubated with the iTRAQ reagents at 25 ^o^C for 2 h. The labeled peptides were dried using vacuum centrifugation, re-solubilized in buffer A consisting of 25 mM NaH_2_PO_4_ in 25% acetonitrile at pH 2.7, and pooled to a combined volume of 4 mL prior to fractionation.

### Peptide fractionation

Fractionation of the peptides was conducted on a Shimadzu LC-20AB liquid chromatography (LC) system (Shimadzu Scientific Instruments, Columbia, USA) equipped with a 4.6 mm × 250 mm UltremexSCX column. After sample loading, separation was performed by eluting at a constant flow rate of 1 mL/min with the following sequence: i) 5% of buffer B (25 mM NaH_2_PO_4_ and 1 M KCl in 25% acetonitrile, pH 2.7) for 7 min, ii) a linear gradient of buffer B from 5% to 60% over 20 min, iii) a linear gradient of buffer B from 60% to 100% over 2 min, iv) 100% of buffer B for 1 min, and v) 5% of buffer B for 10 min. Peptide elution was monitored based on UV absorbance at 214 nm. A total of 20 peptide-containing fractions were collected, desalted on a Strata-X column (Phenomenex, Torrance, USA), lyophilized and stored at -80 ^o^C prior to subsequent mass spectrometric (MS) analysis.

### Peptide detection and identification

Peptide detection and identification was based on the LC-electrospray ionization (ESI)-MS/MS method. Peptide mixtures in each fraction were re-solubilized in buffer A of 5% acetonitrile containing 0.1% formic acid to a final concentration of roughly 0.5 μg/μL. Insoluble components were removed by centrifuging at 20000 g for 10 min. A total volume of 5 μL sample was injected into a trap column (Chrom XP C18m, 3 μm, 120 Å, Eksigent Technologies, Dublin, USA) on a Shimadzu LC-20AD system (Shimadzu Scientific Instruments, Columbia, USA) at a flow rate of 8 μL/min with a mobile phase consisting of 2% acetonitrile and 0.1% formic acid. The sample was washed with the abovementioned mobile phase at a flow rate of 2 μL/min and then the system was switched into line with the analytic column (3C18-CL-120, 3 μm, 120 Å, Eksigent Technologies, Dublin, USA). Separation was performed with the following elution sequence: i) 5% of buffer B (0.1% formic acid in 95% acetonitrile) for 5 min, ii) a linear gradient of buffer B from 5% to 35% over 35 min, iii) a linear gradient of buffer B from 35% to 60% over 5 min, iv) a linear gradient of buffer B from 60% to 80% over 2 min, and v) 5% of buffer B for 10 min. The flow rate was maintained at 300 nL/min throughout the sequence. The eluent was then analyzed on a TripleTOF 5600 system AB SCIEX LP, Concord, Canada) with a Nanospray III source (AB SCIEX LP, Concord, Canada) and a quartz tip (New Objectives, Woburn, USA) using the following key parameters: spray voltage 2.5 kv, nitrogen pressure 30 psi, gas pressure 15 psi, capillary temperature 150 ^o^C. Scanning mode was set to reflectron and resolution to 30000. Survey scans were acquired in 250 ms and 30 product ion scans with a charge state of 2+ to 5+ and exceeding a threshold of 120 counts per second were collected. The cycle time, the Q2 transmission window. Four time bins were summed for each scan at a pulser frequency of 11 kHz and through monitoring of the 40 GHz multichannel time-to-digital converter detector. Collision energy was set to 35 ± 5 eV. Dynamic exclusion was set to half of peak width (15 sec).

### Data analysis

Protein identification was performed using Proteinpilot (version 4.5, AB SCIEX LP, Concord, Canada). Mass spectrum files were converted to the MGF format, which was then used in search against the *Gallus gallus* proteome available from the Uniprot database (www.uniprot.org, proteome ID UP000000539, updated in September, 2016 with a total of 24376 proteins). The following search parameters were used: digestion enzyme—trypsin, MS/MS tolerance—0.1 Da, peptide tolerance—0.05 Da, max missed cleavages allowed—1, fixed modifications—carbamidomethylation on Cysteine and iTRAQ8plex on N-termini as well as lysine, variable modifications—glutamine > pyro-glutamate on N-terminal glutamine), oxidation on methionine and iTRAQ8plex modification on tyrosine, mass value–monoisotopic. To eliminate false positives, only peptides with a score above 20/55 at the 99% confidence interval were counted and each confident protein identification contained at least one unique peptide.

### Bioinformatic analysis

Protein quantification was performed using Proteinpilot (version 4.5, AB SCIEX LP, Concord, Canada). The ratio data were normalized based on the median values. A protein was considered differentially expressed only when its fold change was greater than 1.2 and P-value was lower than 0.05. For functional analysis, the differentially expressed proteins were searched against the following databases: the Gene Ontology database (GO, http://www.geneontology.org), the Kyoto Encyclopedia of Genes and Genomes (KEGG, http://www.genome.jp/kegg/) and the Clusters of Orthologous Groups database (COG, http://www.ncbi.nlm.nih.gov/COG/).

## Results and discussion

### Treatment with TPPPS could enhance the immune activity of chicken peripheral blood lymphocytes

**Fig 1:**
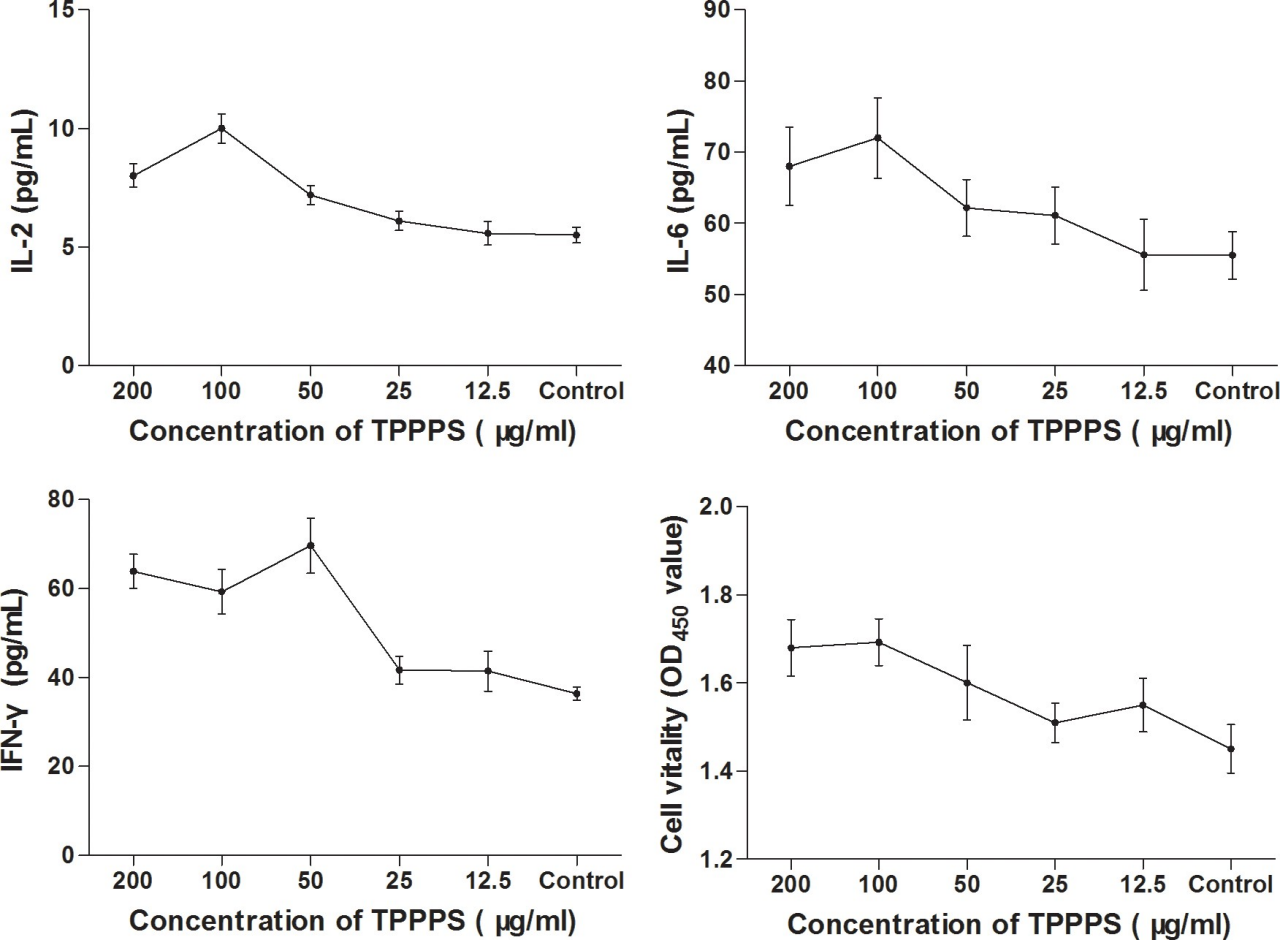
Effects of TPPPS on IL-2, IL-6, IFN-γ secretion and cell vitality in vitro. Activated peripheral blood lymphocyte in 96-well plates were treated with different concentrations of TPPPS, then IL-2, IL-6 and IFN-γ in the supernatant was detected by ELISA. Peripheral lymphocyte vitality was detected by Cell Counting Kit-8. PBS treatment wells served as the control, and the values are presented as mean ± SD of five independent experiments.

Our previous studies have demonstrated that TPPPS could exert an immunostimulatory effect in chickens [14]. To confirm this, we treated chicken peripheral blood lymphocytes with varying concentrations of TPPPS and measured the levels of several inflammatory markers such as IL-2, IL-6 and IFN-γ. As illustrated in Fig 1, the secretory levels of the three cytokines all increased with TPPPS dosage. However, boosting the concentration of TPPPS from 100 μg/mL to 200 μg/mL led to a significant decline in the level of IL-2 and no further stimulation of IL-6 or IFN-γ production. Overall, treatment with 100 μg/mL of TPPPS demonstrated the greatest effect on the activation of chicken peripheral blood lymphocytes.

### iTRAQ proteomic analysis of TPPPS-treated chicken peripheral blood lymphocytes

**Fig 2:**
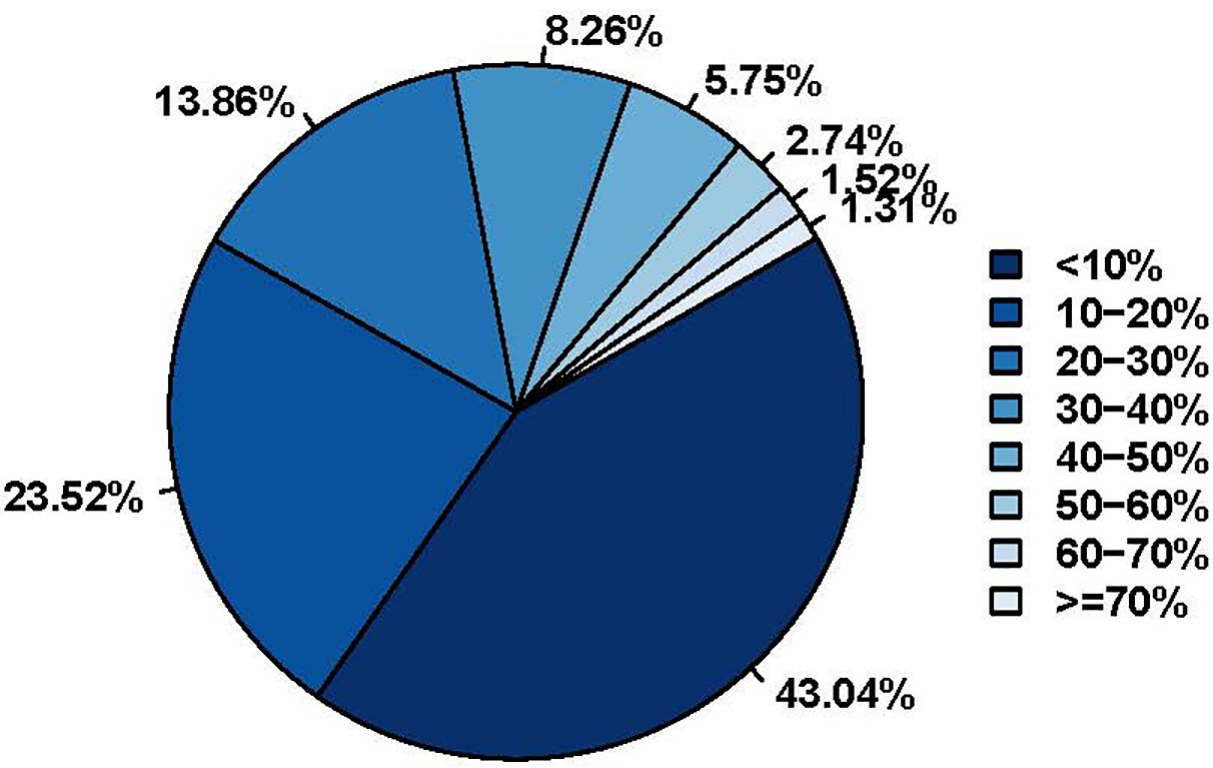
Distribution of identified protein candidates according to their peptide coverage rates. The percentages of peptide coverage are arranged vertically in an ascending order on the right. The color of the box to the left of each peptide coverage percentage value matches that of one of the slices in the pie chart. The percentage value next to each slice of the pie chart represents the percentage of protein candidates with the coverage rate specified by the color of the said slice.

We subsequently compared the proteome of chicken peripheral blood lymphocytes treated with 100 μg/mL of TPPPS to that of untreated cells using the iTRAQ technique. Overall, we identified a total of 3244 proteins (Table 1) from 20034 detected unique peptides in 96336 highly confident spectra (>95% confidence). Among the identified proteins, 2636 contained at least two unique peptides each. The average length of the identified peptides was calculated to be 14.76 with those composed of 9–17 amino acids being the most abundant (S1 Fig), which was in line with other iTRAQ proteomic studies [15]. Sequence coverage reflects the confidence level of protein identification and thus serves as a useful indicator of the reliability of iTRAQ proteomics method. As illustrated in Fig 2, 43.04%, 23.52% and 33.44% of all identified protein candidates showed sequence coverage rates of <10%, 10%-20% and >20%, respectively. The average protein sequence coverage was around 17.83%.

**Table 1:** Numbers of differentially expressed proteins from peripheral blood lymphocyte treated with TPPPS.

Type	TPPPS/Blank
**Total number of identified proteins**	3244
**The number of significantly up-regulated proteins**	3
**The number of significantly down-regulated protein quantity**	7

Note: A protein is considered significantly up-regulated if its fold-change between the two experiment groups exceeded 1.2 and the P value was no more than 0.05. Similarly, significant down-regulation is defined by fold-change ≤ 0.82 and P ≤ 0.05.

**Fig 3:**
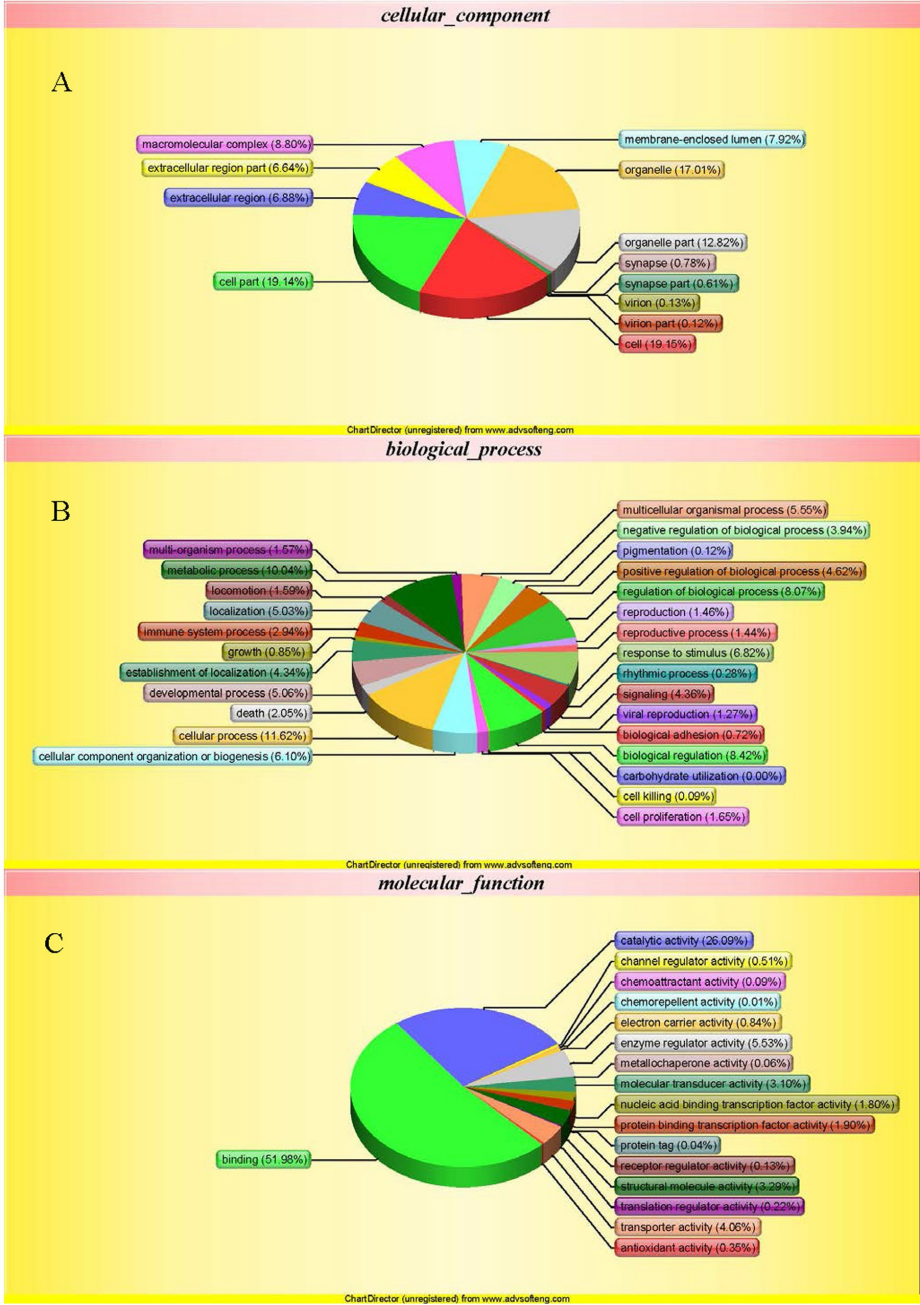
Gene ontology (GO) analysis of proteins in chicken peripheral blood lymphocytes treated with TPPPS. The differentially expressed proteins were classified into cellular component (A), biological process (B), and molecular function (C) according to the GO terms.

**Fig 4:**
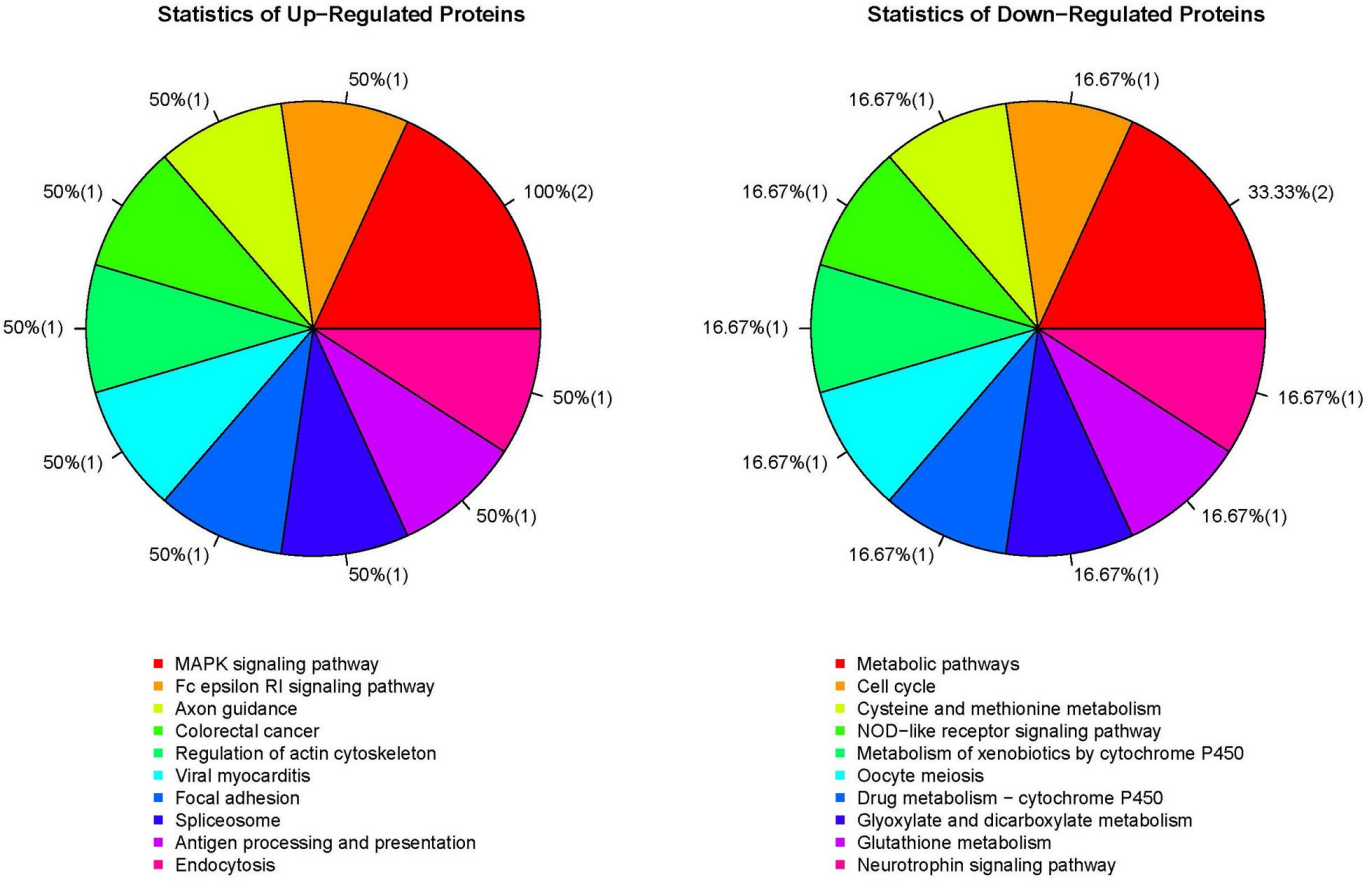
KEGG pathway analysis of the differential proteins identified from TPPPS-treated chicken peripheral blood lymphocytes. The number in each pair of parentheses denotes the number of differentially expressed protein candidates whose KEGG annotation matches that specified by the slice.

Examination of all confidently identified proteins using the criteria of fold-change > 1.2 or <0.82 and P < 0.05 showed that three candidates were up-regulated and seven were down-regulated in TPPPS-treated lymphocytes compared to the controls (Tables 1 and 2). Functional analysis of the differentially expressed proteins was performed using GO, KEGG and COG databases. The main enriched GO functions that could be meaningfully interpreted and related to the current study included response to stimulus, immune system process, cell proliferation, biological adhesion, death and cell killing (Fig 3). KEGG analysis suggested that the differentially expressed proteins were involved in several pathways that could play important roles in cell survival and apoptosis, cellular redox homeostasis, cell cycle and immune response (Fig 4). Interestingly, Ras suppressor protein 1 (RSU1), which was down-regulated in TPPPS-treated lymphocytes, were found to be a member of the Nod-like receptor (NLR) signaling pathway implicated in the regulation of innate immune response.

**Table 2:** List of the differential proteins with Uniprot accession ID, names and annotations.

Accession	Name	Fold change	Diff_state	Function
Q5F337	RAC2	1.67	up	Protein kinase regulator activity
P17785	ANXA2	1.42	up	Vesicle transport, apoptosis and inflammation
F1NWP3	HSPA8	1.29	up	Protein folding and quality control
F1NMC3	MTHFD1	0.73	down	Tetrahydrofolate synthase
E1C245	Uncharacterized protein	0.72	down	
E1BX85	RSU1	0.62	down	Ras signal transduction
E1BWI3	GSTO1	0.62	down	Dehydroascorbate reductase activity
F1P284	LTA4H	0.56	down	Conversion of leukotriene A4 to leukotriene B4 in arachidonic acid metabolism
Q5ZMD1	14-3-3 protein theta	0.45	down	Adapter protein with regulatory Roles in a wide range of pathways
E1C489	ENOPH1	0.45	down	Catalyzes DK-MTP-1-P

Our current study provided valuable mechanistic insights into the immunostimulatory effects of TPPPS on chicken peripheral blood lymphocytes. Consistent with our previous studies, incubation of the lymphocytes with 100 μg/mL of TPPPS was found to increase the secretion levels of several pro-inflammatory cytokines such as IL-2, IL-6 and IFN-γ. Subsequent proteomic analysis of both TPPPS-treated and untreated lymphocytes led to the identification of 10 differentially expressed proteins from a total of 3355 confidently detected proteins. The size of our data set was similar to those published in other proteomic studies using the iTRAQ labeling technique. For example, Ren and colleagues reported 21 up-regulated and nine down-regulated candidates based on 961 confidently detected proteins and 2863 unique peptides derived from the proteomes of five different tissues in growing *Portunus trituberculatus* [16]. Similarly, Cao el al. investigated the proteomic changes that occurred in sweet oranges as a result of *Candidatus Liberibacter asiaticus* infection and identified 30 differentially expressed candidates from 686 unique proteins [17]. Further analysis of the proteins with aberrant expression levels in TPPPS-treated lymphocytes revealed associations with immune response, cell apoptosis, cell cycle and redox homeostasis, suggesting that changes in these functions might underlie the immunostimulatory properties of the polysaccharide.

Heat shock proteins (Hsp) are a group of highly conserved proteins that act as molecular chaperones and cellular housekeepers. Recently, there is increasing evidence that Hsps possess immunogenic properties and are involved in the regulation of immune response [18]. Enhanced expression of Hsps has been associated with augmented immune activity in host systems against infection, possible due to the fact that they are often abundant in pathogens and well conserved across different species [19,20]. Hsp70 has been reported to exhibit adjuvant activity by promoting adaptive immune response to antigenic cells [21]. Xu et al.’s proteomic study showed that Hsp70 and Hsp 90-beta were up-regulated and down-regulated, respectively, in intestinal tissues harvested from mice fed with *Lentinula edodes*-derived polysaccharides compared to the control group that received a normal diet [22]. Interestingly, the authors also observed a lower level of Annexin A2 in the polysaccharide group, which mirrored the finding in our current study. Similarly, Xu and coworkers found that treatment of chickens with polysaccharides of *Atractylodes macrocephala Koidz*. led to a higher level of Hsp70 and Hsp27 in the spleen [23]. Taken together, these results implied that Hsps could play an important role in the immunostimulatory effect of plant- and fungus-derived polysaccharides.

LTA4H is a bifunctional hydrolytic enzyme that catalyzes the conversion of leukotriene A4 to leukotriene B4 (LTB4), the latter of which is implicated in both pro- and anti-inflammatory pathways [24]. In addition, LTB4 has also been shown to promote leukocyte accumulation and phagocytosis, as well as mediate the recruitment of effector T cells [25]. Furman et al. compared the gene expression profiles between 53 females and 34 males immunized with influenza vaccine, which revealed that several lipogenesis-related genes, including LTA4H, were up-regulated in the male participants [26]. The authors suggested that these differentially expressed lipogenic genes could be responsible for the reduced response of the male subjects to the vaccination. In another study, increased expression of LTB4 and IFN-γ promoted the recruitment of memory T cells in a murine model of *Histoplasma capsulatum* infection [27]. In our study, chickens administered with TPPPS exhibited decreased LTB4 expression compared to the untreated control, which reflected the anti-inflammatory role of the enzyme.

Toll-like receptors (TLR) and NLRs are two major groups of proteins responsible for the regulation of innate immune response [28]. In our study, we identified several TLR- or NLR-related proteins that showed altered expression patterns in TPPPS-treated chickens. For example, 14-3-3 protein theta has recently been discovered as a component of the TLR signaling pathway and was shown to regulate the expression of pro-inflammatory cytokines such as IL-6, IL-8 and Tumor Necrosis Factor (TNF)-α [29]. RAC2 is a key component of nicotinamide adenine dinucleotide phosphate (NADPH) oxidase (NOX) [30], which has been reported to interact with TLR4 to enhance the activity of nuclear factor (NF)-κB [31]. NOX-induced production of reactive oxygen species also significantly contributes to host defense against pathogen invasion [32]. Similarly, our functional analysis has suggested the involvement of RSU1 in the NLR signaling pathway. These findings are consistent with a large body of literatures that indicate a role of vaccine adjuvants in triggering and augmenting the innate immune response in hosts [33]. In fact, the acetate derivative of inulin, a polysaccharide, from dahlia tubers, demonstrated pronounced TLR-4 agonistic activity in peripheral blood lymphocytes from several different animal species [34]. Therefore, it is plausible that TLR and NLR signaling pathways might also mediate the stimulation of host innate response by TPPPS.

### Conclusion

In summary, we presented the first detailed and comprehensive proteomic study of the peripheral blood lymphocytes in chicken administered with TPPPS. Compared to the untreated control, our profiling revealed that several of the differentially expressed proteins in TPPPS-treated chickens were implicated in pathways related to host innate immune response, stress-induced immune response, and/or lipid synthesis. These results could pave the way for further research on the molecular mechanism that underlies the immunostimulatory effect of TPPPS.

## Supporting information

S1 Fig Distribution of detected peptides according to their lengths. (TIF)

S1 Table Summary of identified differential protein candidates in this study. (DOCX)

S2 Table Summary of identified proteins related to Gene ontology (GO) analysis in this study. (XLSX)
