# Supplementary figures and images for: Proteomics analysis of chicken peripheral blood lymphocyte in Taishan Pinus massoniana pollen polysaccharide regulation
Source: PLoS One. 2018 Nov 29;13(11):e0208314. doi: 10.1371/journal.pone.0208314 (PMC6264863; doi:10.1371/journal.pone.0208314)

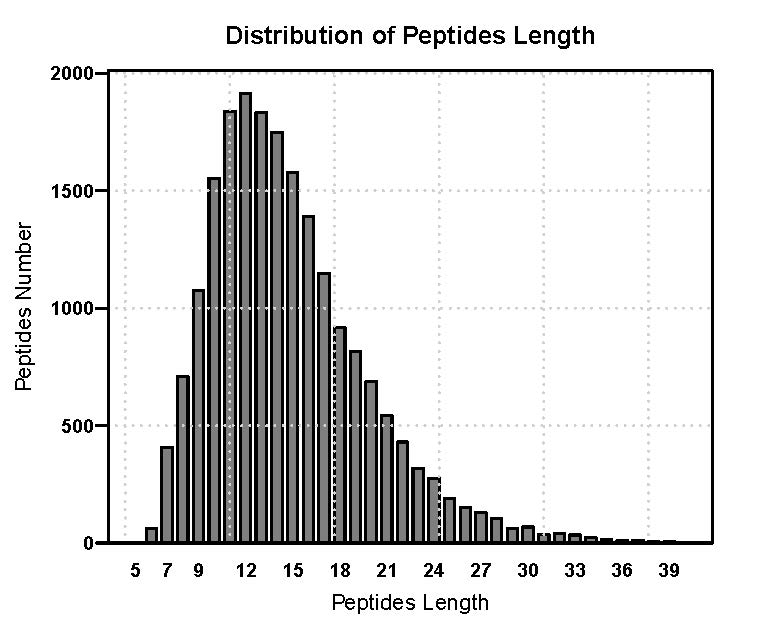

Supplement: S1 Fig — (TIF) [file pone.0208314.s001.tif]
