# Supplementary material for: Proteomics analysis of chicken peripheral blood lymphocyte in Taishan Pinus massoniana pollen polysaccharide regulation
Source: PLoS One. 2018 Nov 29;13(11):e0208314. doi: 10.1371/journal.pone.0208314 (PMC6264863; doi:10.1371/journal.pone.0208314)
Supplement: S1 Table — (DOCX) [file pone.0208314.s002.docx]

**Supporting Information**

**S1 Fig. Distribution of detected peptides according to their lengths**

**S1 Table. Summary of identified differential protein candidates in this study**

| **Accession** | **Name** | **Protein Length** | **of Peptides** | **%Cov(95)** | **#of Spectra** | **Function** | **Fold change** | **Diff_state** |
| --- | --- | --- | --- | --- | --- | --- | --- | --- |
| Q5F337 | RAC2 | 192 | 7 | 27.6 | 41 | Protein kinase regulator activity | 1.67 | up |
| P17785 | ANXA2 | 339 | 30 | 75.81 | 305 | Vesicle transport, apoptosis and inflammation | 1.42 | up |
| F1NWP3 | HSPA8 | 646 | 40 | 59.43 | 677 | Protein folding and quality control | 1.29 | up |
| F1NMC3 | MTHFD1 | 935 | 18 | 26.63 | 45 | Tetrahydrofolate synthase | 0.73 | down |
| E1C245 | Uncharacterized protein | 5411 | 101 | 24.51 | 289 |  | 0.72 | down |
| E1BWI3 | RSU1 | 277 | 11 | 50.54 | 124 | Ras signal transduction | 0.62 | down |
| E1BX85 | GSTO1 | 239 | 11 | 47.28 | 43 | Dehydroascorbate reductase activity | 0.62 | down |
| F1P284 | LTA4H | 612 | 22 | 41.5 | 137 | Conversion of leukotriene A4 to leukotriene B4 in arachidonic acid metabolism | 0.56 | down |
| Q5ZMD1 | 14-3-3 protein theta | 245 | 19 | 61.63 | 126 | Adapter protein with regulatory Roles in a wide range of pathways | 0.45 | down |
| E1C489 | ENOPH1 | 261 | 6 | 29.89 | 9 | Catalyzes DK-MTP-1-P | 0.45 | down |
